# Multi-pass probing for high-sensitivity tomographic interferometry

**DOI:** 10.1038/s41598-021-94436-6

**Published:** 2021-07-23

**Authors:** Stefan Karatodorov, Roberto Lera, Marek Raclavsky, Sebastian Lorenz, Uddhab Chaulagain, Jaroslav Nejdl

**Affiliations:** 1 ELI Beamlines Center, Institute of Physics ASCR, 252 41 Dolní Břežany, Czech Republic; 2 FNSPE, Czech Technical University in Prague, 115 19 Prague, Czech Republic

**Keywords:** Optical techniques, Imaging and sensing

## Abstract

Optical probing is an indispensable tool in research and development. In fact, it has always been the most natural way for humankind to explore nature. However, objects consisting of transparent materials with a refractive index close to unity, such as low-density gas jets, are a typical example of samples that often reach the sensitivity limits of optical probing techniques. We introduce an advanced optical probing method employing multiple passes of the probe through the object to increase phase sensitivity, and relay-imaging of the object between individual passes to preserve spatial resolution. An interferometer with four-passes was set up and the concept was validated by tomographic characterization of low-density supersonic gas jets. The results show an evident increase of sensitivity, which allows for the accurate quantitation of fine features such as a shock formed by an obstacle or a barrel shock on the jet boundary in low ambient gas pressures. Despite its limitations in temporal resolution, this novel method has demonstrated an increase in phase sensitivity in transmission, however, it can also be employed to boost the absorption or polarization contrast of weakly interacting objects in both transmission and reflection setups, thus, upgrading the sensitivity of various optical characterization methods.

Optical interferometry is a family of probing techniques that utilize the interference between optical waves imprinted in the phase of the probing beam for precise measurement of small distances, observation of refractive index variations, visualization of surface irregularities, determination of wavelengths, and many other characterization methods^1^. Science and industry foster an ever-growing demand for high-precision measurements. This is the driving force for improving the capabilities of existing optical probing methods and for the development of new interferometer designs.

One of the modern scientific fields which requires precise interferometric measurements is underdense laser-plasma interaction. This is used for fundamental research, generation of compact X-ray sources^2,3^, or for laser wakefield electron acceleration (LWFA)^4–7^. In this area, laser targets in the form of gas cells, capillaries, or gas jets with various density profiles are routinely used. The gas jets employed for LWFA in particular, range from axisymmetric types generated by cylindrically symmetric nozzles to complex non-rotationally symmetric jets produced by nozzles of various shapes or by gas streams perturbed by obstructions (razor blades, wires), which generate gas distributions tailored to specific purposes, e.g. pressure ramps and/or shocks^8–12^. The use of such complex gas jet distributions allows for better control over the laser-plasma parameters, which results in electron acceleration with improved shot-to-shot stability, increased bunch charge, smaller energy spread, etc. Thus, the precise knowledge of the gas medium, in which the laser propagates, is essential for the optimization of the laser-plasma interaction processes as well as for benchmarking numerical codes that model the interaction.

Interferometry is the most popular method for assessing the density distribution of gas jets^13,14^. Lately, the research on interferometric techniques for neutral gas jet density characterization focuses on the improvement of interferometric sensitivity, e.g. by the use of multiple passes through the gas target. A Michelson interferometer with up to 8-passes^15^ shows an increase in phase sensitivity, but limits its applicability to axially symmetric objects with small refractivity and low requirements on spatial resolution. Relay imaging in the object arm of a double-pass Michelson interferometer introduced in^16^ allowed increase of sensitivity without affecting the high spatial resolution of the method. Another trend is the use of tomography for three-dimensional reconstruction of tailored gas density distributions^17–20^. With advances in high-power laser technology, the characterization of LWFA targets becomes increasingly challenging, as either their density decreases^21^ or their dimensions are reduced^22^. Both trends decrease the phase shift of the probe beam, often reaching the sensitivity limits of the characterization method, which is even more critical if the working gas refractive index approaches unity, such as in the case of helium.

In this article, we introduce a new probing method with increased sensitivity and near diffraction-limited spatial resolution. The method is adapted for shearing interferometry and employed for gas jet density characterization. The distinctive characteristic of our interferometer is the multiple passes of the probing beam through the gas target facilitated by relay-imaging arms that image the object on itself. Using this setup, we can substantially increase the phase sensitivity of the device due to the increased number of passes through the medium and, at the same time, the relay-imaging arms preserve the spatial information undistorted. Additionally, an increase in sensitivity is achieved by using a shorter wavelength (405 nm) of the probe beam compared to the conventionally used He–Ne laser (633 nm).

The multi-pass interferometer was employed for a study of low-density gas jets typically used for LWFA. Two different gases (He, Ar) at diverse backing pressures were studied. We have demonstrated an increased phase sensitivity of our method by comparing the results of probing the density distribution of an axisymmetric gas jet with double-pass and four-pass configurations to the standard single-pass configuration. We were also able to perform a high-quality tomographic reconstruction of a non-rotationally symmetric gas jet with a shock in both He and Ar jets with low backing pressures. Furthermore, we have observed the onset of a barrel shock formation during the expansion of the gas jet in a non-zero ambient gas environment that can take place in the case of high-repetition-rate operation of a gas jet.

## Multi-pass interferometry with relay-imaging arms

The new concept presented in this article consists of a multi-pass probing setup coupled to a shearing Wollaston interferometer. The unique feature of this setup is the capability to work with a different number of passes through the object (one, two, or four), using passive optical elements only, while preserving the spatial resolution. These capabilities are achieved by employing relay-imaging object arms and, in the case of four-pass probing, polarization switching of the probe beam. The increased number of passes allows more phase-shift to be accumulated, thus enabling phase sensitivity increase, while the high spatial resolution of the optical setup allows for precise tomographic measurements of complex phase objects.

### Double-pass configuration

**Figure 1:**
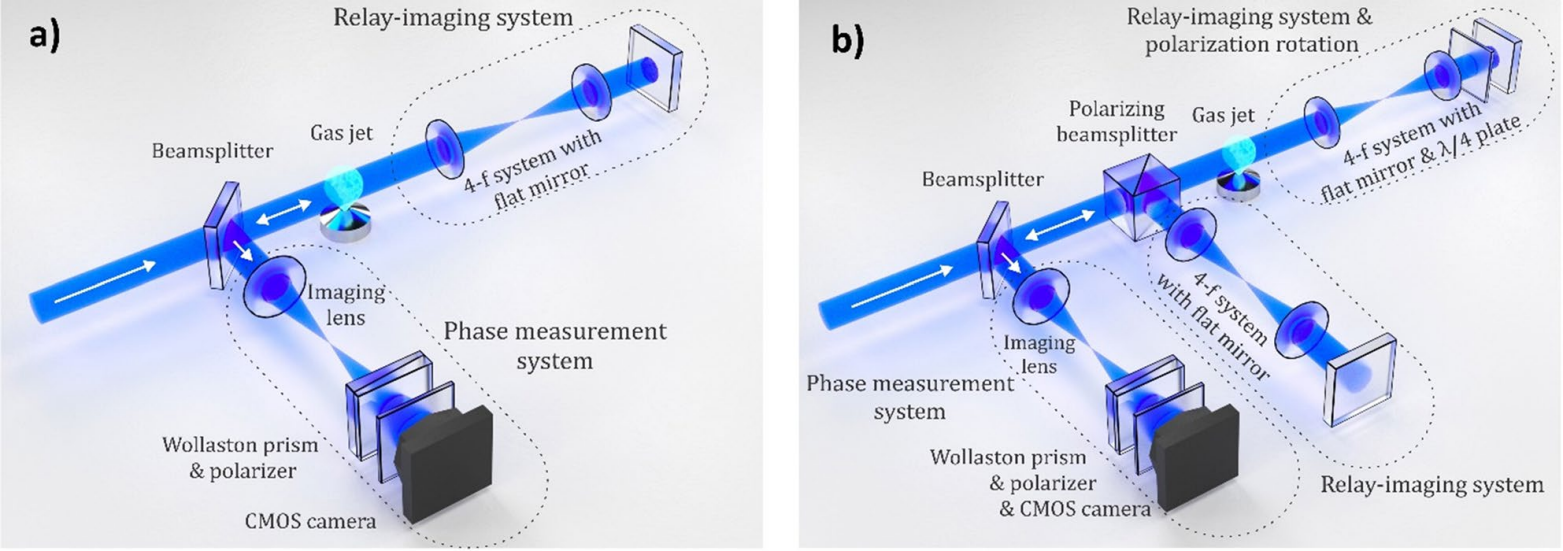
Schematics of the double-pass setup (**a**) and four-pass setup (**b**) for interferometric gas jet characterization. The first reflections of the beam on the beam splitters and their beam blocks are not shown.

A schematic of the double-pass interferometry setup is shown in Fig. 1a. First, the collimated 405 nm laser beam is partially transmitted by a beam splitter and travels towards the vacuum chamber (not shown in the figure) illuminating the gas jet. Then the beam travels to the relay-imaging optical system that enables a second pass through the gas jet while preserving the spatial information undistorted. The beam is then reflected by the beam splitter towards the Wollaston interferometer, where the phase distortion of the beam is measured.

### Four-pass configuration

We have employed polarization switching with two relay-imaging arms to facilitate four passes through the gas jet target (Fig. 1b). In this configuration, the beam travels through the beam splitter and a polarizing beam splitter and illuminates the gas jet. Similarly, to the double-pass configuration the relay-imaging arm is used to enable a second pass, but also a quarter-wave plate is inserted in the collimated beam with fast axis oriented under an angle of 45 deg with respect to the incoming polarization to perform switching of the linear polarization (from horizontal to vertical). Because of the changed polarization, the beam is subsequently reflected by the polarizing beam splitter to the second relay-imaging arm of the setup and probes the jet for the third time before it enters the first relay-imaging arm again. This arm ensures the fourth interaction of the probe with the jet and switches the polarization back to horizontal. The horizontally polarized beam is then transmitted through the polarizing beam splitter and afterwards it is reflected by the non-polarizing beam splitter towards the phase measurement system.

In this configuration, the first and the fourth pass of the probe through the gas jet are performed with horizontally polarized light while the second and third passes are with vertical polarization. This would be significant if the probed object was anisotropic, but it should not affect the characterization of gas jets, as those are considered optically isotropic and each of the passes contributes to the phase distortion of the probe equally. Moreover, the four-pass configuration can be easily switched to the double-pass one by rotating the quarter-wave plate by 45 degrees. This disables the polarization switching and eliminates the second relay-imaging arm.

### Single-pass configuration

In order to compare the results obtained with the multi-pass probing schemes to the standard single-pass setup, we inserted a turning mirror between the gas jet and the first relay-imaging system and sent the collimated probe beam from the other side of the jet. Thus, the probe beam interacted with the jet only once, and all the optical components in the phase measurement part of the setup remained unchanged.

## Results

### Density profiles of axisymmetric gas jets

**Figure 2:**
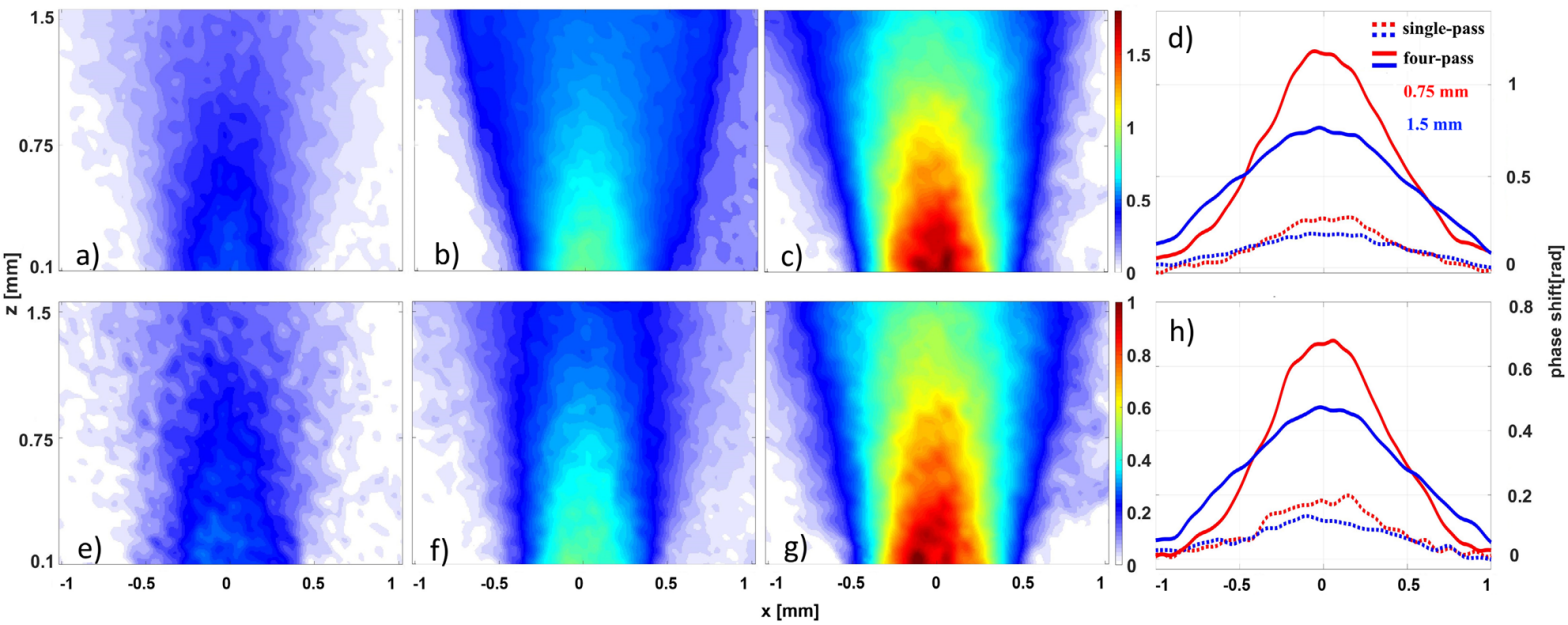
Comparison of spatial phase distributions obtained at a different number of passes of the probe beam through the gas jet generated by a cylindrical nozzle for backing pressure of 1.6 bar Ar (**a**–**c**) and 7 bar He (**e**–**g**). For the phase maps from left to right single-pass, double-pass, and four-pass. Phases at two different heights above the nozzle in the single-pass and the four-pass configurations are shown for Ar (**d**) and He (**h**) jets.

The performance of the interferometric setups with different numbers of passes is demonstrated by comparing the phase maps of axisymmetric super-sonic gas jets shown in Fig. 2. The phase maps obtained by a continuous wavelet transform (CWT) method (see Methods for details) correspond to Ar with 1.6 bar backing pressure (a–c) and He with 7 bar backing pressure (d–f). The data analysis reveals that increasing the number of passes through the medium corresponds to an increase of the accumulated phase shift by the same factor. In order to quantify the sensitivity of the multi-pass interferometer, we have evaluated the background noise level for the single-pass, two-pass, and four-pass configurations. The background noise is calculated from a standard deviation of the phase shift in an area of 1 × 1 mm^2^ in the corner of the phase map, where we don’t expect any contribution of the gas. We obtain the phase standard deviation of 20 mrad for all configurations as shown in Table 1, where maximum accumulated phases are also listed.

**Table 1:** Comparison of maximum phase shifts and background noise in radians for different probing setups.

Probing configuration	1 pass	2 pass	4 pass
Maximum signal 7 bar He	0.26	0.48	1.00
Maximum signal 1.6 bar Ar	0.44	0.88	1.79
Background noise	0.02	0.02	0.02

We can define the sensitivity of the given setup in terms of variation of the area density $\sigma_{N}^{{\left( {{\text{gas}}} \right)}} = {\Delta }n \times L$ as per^16^, where ${\Delta }n$ is the uncertainty of the gas number density, *L* is the length of the gas medium, and *N* denotes the number of passes. In the case of Ar, we obtain sensitivities of $\sigma_{1}^{{\left( {{\text{Ar}}} \right)}} = 1.2 \times 10^{16} \;{\text{cm}}^{{ - 2}}$, $\sigma_{2}^{{\left( {{\text{Ar}}} \right)}} = 0.6 \times 10^{16} \;{\text{cm}}^{{ - 2}}$, and $\sigma_{4}^{{\left( {{\text{Ar}}} \right)}} = 0.3 \times 10^{16} \;{\text{cm}}^{{ - 2}}$ for a single-pass, two-pass, and four-pass setup, respectively, and in the case of He we get, $\sigma_{1}^{{\left( {{\text{He}}} \right)}} = 1 \times 10^{17} \;{\text{cm}}^{{ - 2}}$, $\sigma_{2}^{{\left( {{\text{He}}} \right)}} = 5 \times 10^{16} \;{\text{cm}}^{{ - 2}}$, and $\sigma_{4}^{{\left( {{\text{He}}} \right)}} = 2.5 \times 10^{16} \;{\text{cm}}^{{ - 2}}$ for a single-pass, two-pass, and four-pass setups, respectively. Thus, with the four-pass setup one can infer the measurable density variations of a 1 mm-thick medium as $3 \times 10^{16} \;{\text{cm}}^{{ - 3}} { }$ in Ar and $2.5 \times 10^{17} \;{\text{cm}}^{{ - 3}}$ in He.

**Figure 3:**
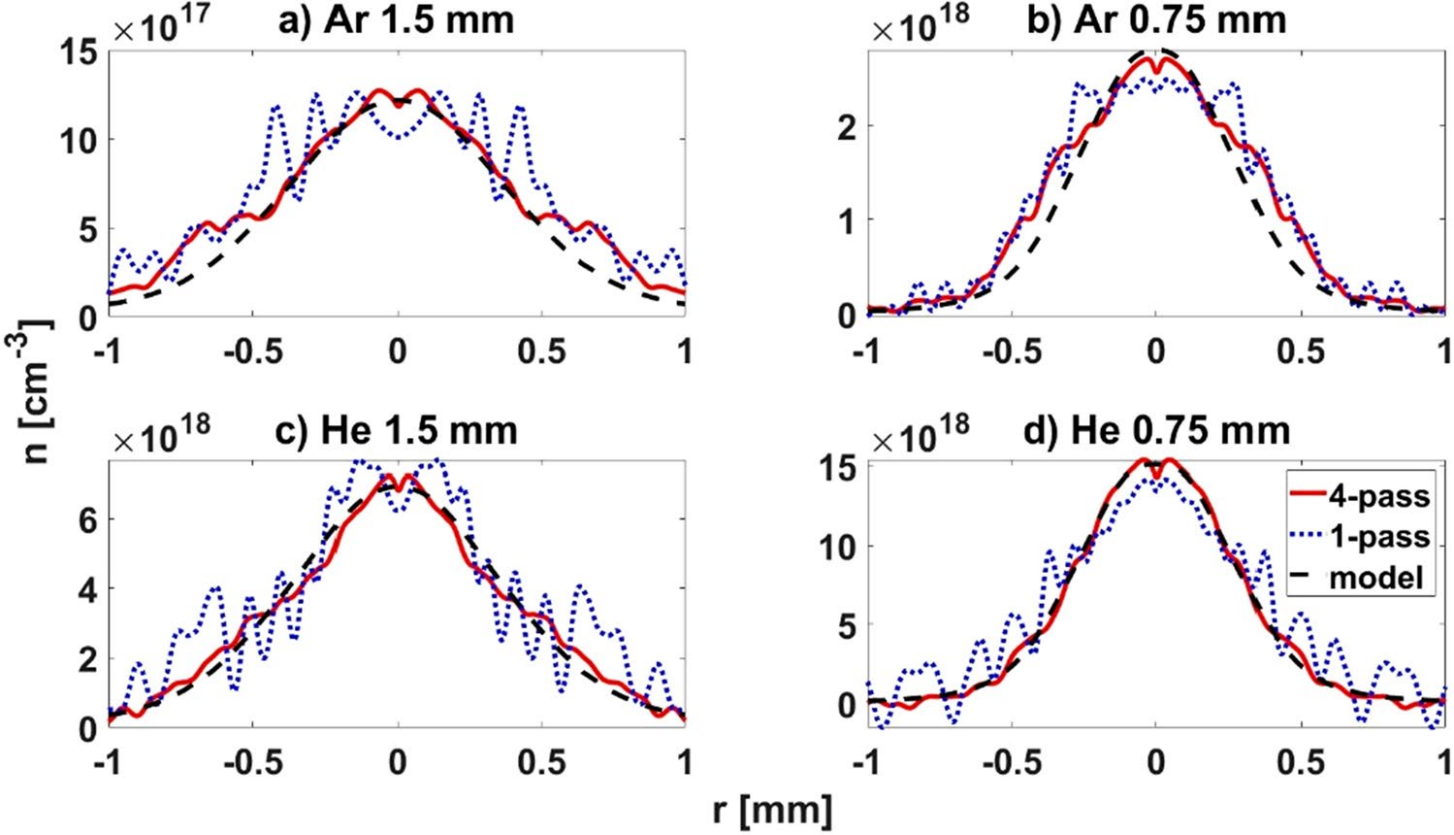
Comparison of density profiles calculated using the inverse Abel transform of an axisymmetric gas jet with backing pressure of 1.6 bar Ar (**a**, **b**) and 7 bar He (**c**, **d**) at 1.5 mm (**a**, **c**) and 0.75 mm (**b**, **d**) above the nozzle exit. The profiles for single-pass (dotted blue lines) and four-pass (solid red lines) probing configuration calculated from the phase maps shown in Fig. 2 are compared to the profiles from the hydrodynamic simulations (dashed black line).

The density distribution of the axisymmetric supersonic gas jets in Ar and He are reconstructed from the phase maps shown in Fig. 2 using the inverse Abel transform. In Fig. 3 the density profiles for single-pass and four-pass configurations are compared at two different heights above the nozzle (0.75 mm and 1.5 mm) for Ar and He. The reconstructions are compared to the results of 3D axisymmetric hydrodynamic simulations of the gas jet in a steady regime. The system of partial differential equations consisting of Navier–Stokes equations for compressible flow, equation of conservation of energy and Shear Stress Transport turbulence model is solved by Finite Volume Method. More details of the simulation method are addressed in^23^. As seen in Fig. 3 there is good agreement of the simulated and measured density values for both heights and gases. The agreement is better in the central part of the jet while at the sides of the jet profiles, the interferometry results show wider jet diameters than the simulations. We ascribe this, mostly to the imperfection of the 3D printing method, by which the used nozzle was fabricated, that could cause variations from the designed nozzle throat dimensions.

It is obvious that increasing the number of passes substantially decreases the noise of the calculated density profiles for both gases; however, this effect is more pronounced for He, because the phase signal-to-noise ratios are lower in that case. Keeping in mind that Abel inversion is a nonlinear transform with a strong dependence on the distance from the axis we performed a quantitative noise analysis that revealed a fivefold decrease of the noise around 1 mm from the jet axis when comparing the He density profiles acquired with the four-pass setup to the ones from the single-pass configuration.

### Density distributions of non-rotationally symmetric gas jets

In many cases, e. g. advanced LWFA schemes^8,11^, characterization of spatially tailored gas density distributions is required. Such gas distributions can be generated by non-rotationally symmetric gas nozzles, e.g. rectangular gas nozzles and/or by an obstruction placed in the gas flow, such as a razor blade or a wire. We have created a non-rotationally symmetric gas jet by placing a razor blade in the flow of the circular supersonic de Laval nozzle and observed the formation of a shock front. The blade was positioned 1 mm above the nozzle output, it was perpendicular to the nozzle axis with a distance of 0.5 mm from this axis (see Fig. 6 in Methods).

**Figure 4:**
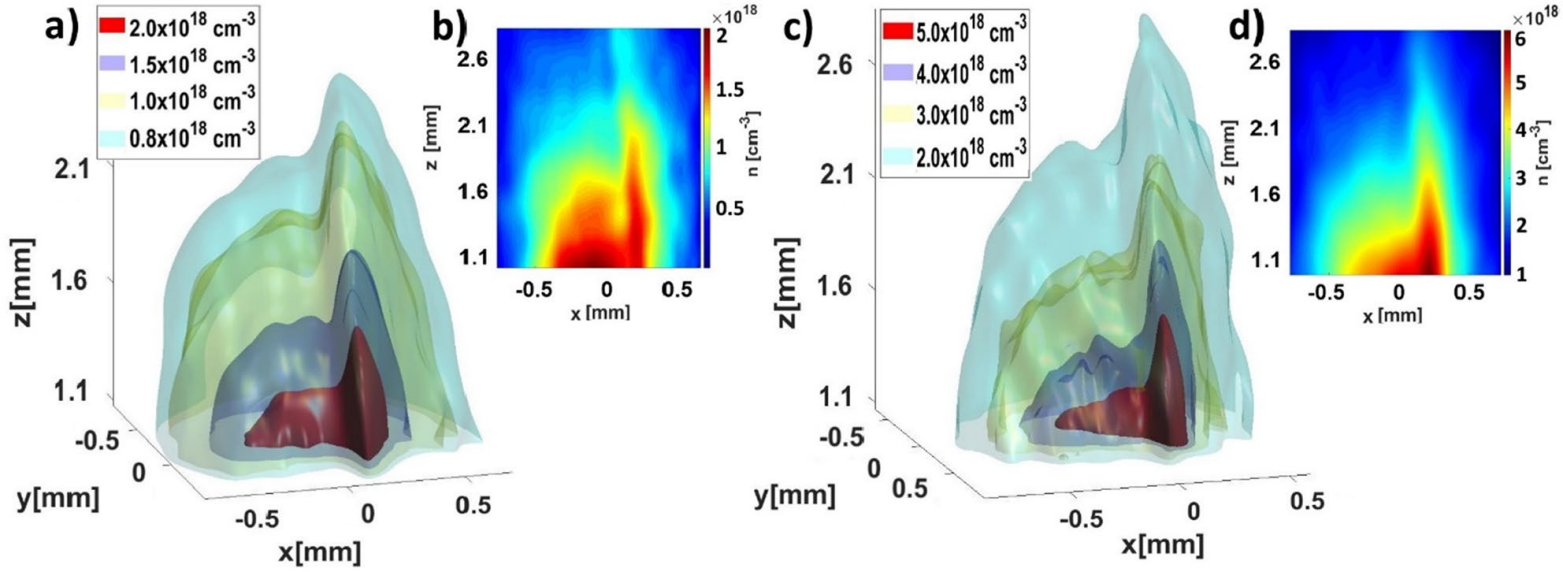
Tomographic density reconstructions of Ar (**a**, **b**) and He (**c**, **d**) gas jets obstructed by a razor blade placed 1 mm above the nozzle at a backing pressure of 1.6 bar and 7 bar, respectively. Gas distribution is represented by density isosurfaces with values shown in the legend. The z-coordinate describes the height above the nozzle. Vertical cross-sections of the density profiles in the middle of the gas jet (plane y = 0) is shown in the insets (**b**) and (**d**) for Ar and He, respectively.

In order to assess the gas density distribution of the non-rotationally symmetric jets, we performed tomographic measurements. Four-pass interferograms of 90 projections at a step of two degrees were acquired by rotating the target. Each interferogram was recorded from a separate shot of the jet. For each projection angle, an interferogram with the jet and without the jet are recorded to enable subtracting the phase distortions caused by the optical system. The interferograms of individual projections were treated with the CWT algorithm to obtain the phase maps, from which the density distribution was reconstructed using the filter back projection (FBP) method. The results of the reconstructed density distributions are shown in Fig. 4 for Ar at backing pressure of 1.6 bar and He at backing pressure of 7 bar. The 3D density profiles represented by density isosurfaces are complemented by corresponding 2D density profiles in the central planes (y = 0) that are shown in the insets.

In both profiles, we can clearly see the two distinct regions of the density distribution—the steep shock with a high-density gradient (on the right) and the smoother plateau of the gas jet (on the left). For Ar gas, we have gas densities of up to 2 × 10^18^ cm^−3^ in the 100-µm wide shock region right above the blade, while for He the density in this region goes up to 6 × 10^18^ cm^−3^. The wide plateau has about 1.5 times lower density compared to the density of the shock in both cases. The two distinctive density regions are connected by a steep density down-ramp. As expected, the density of both features gradually decreases farther away from the blade. Except for the absolute value of the density, the profiles of both gas jets are similar.

### Formation of shock waves during jet expansion in the ambient gas environment

Shocks can be generated not only by inserting an obstacle in the gas stream but also by allowing the supersonic jet to expand in an environment with non-zero ambient gas pressure^24^. The latter can lead to the formation of shock waves, because of the interaction of the expanding gas jet molecules with the ambient environment. The effect is known as barrel shock, due to its characteristic shape^24,25^.

**Figure 5:**
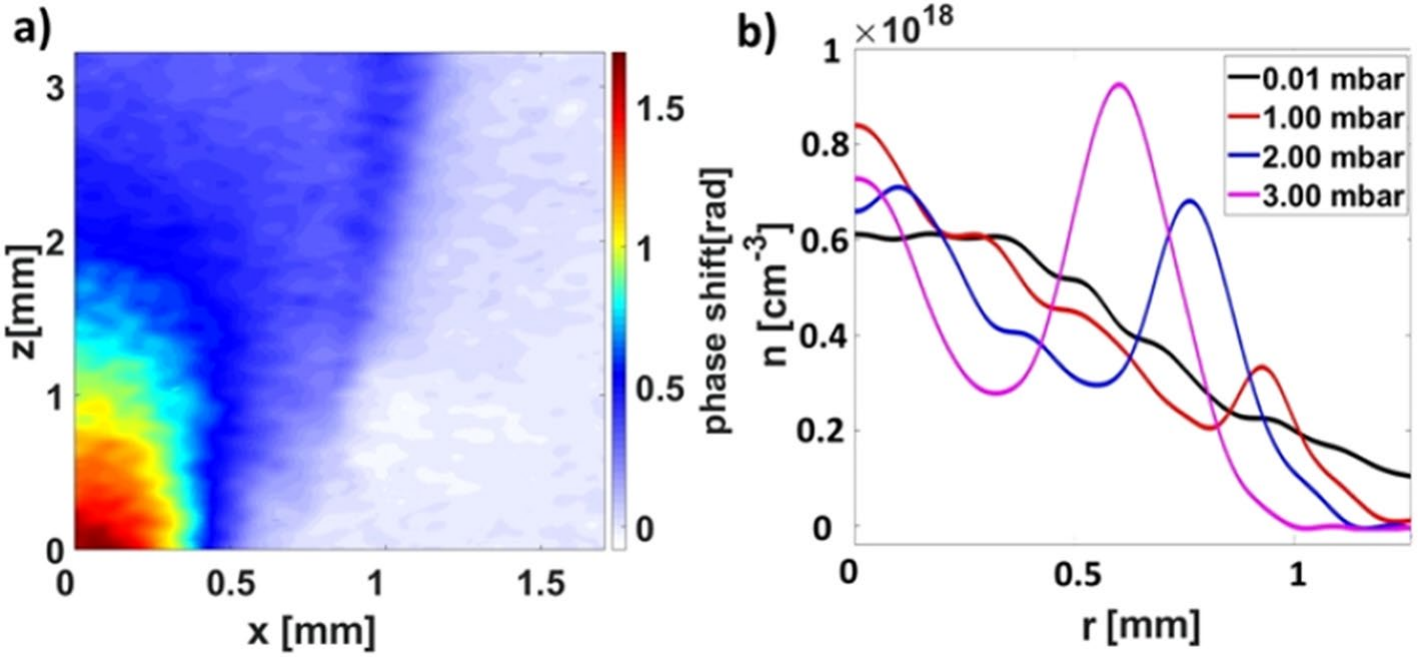
(**a**) Phase map of Ar with backing pressure of 1.6 bar and ambient pressure of 1.0 mbar showing the clear barrel shock formed on the edge of the jet. (**b**) Gas density distribution of Ar gas jets (backing pressure 1.6 bar) during expansion in ambient gas pressure in the range of 0–3 mbar at the height of 2 mm above the nozzle exit.

We studied the onset of barrel shock by generating an axisymmetric gas jet of Ar at 1.6 bar backing pressure in ambient Ar gas environment with pressures in the range of 0.01–3 mbar. The phase map obtained at the particular ambient pressure of 1 mbar is shown in Fig. 5a and the jet density profiles at 2 mm above the nozzle output for various ambient pressures obtained using the inverse Abel transform are plotted in Fig. 5b. While the barrel shock is absent for the lowest ambient pressure, its onset is noticeable at ambient pressure of 1 mbar at a distance of 1 mm from the jet axis (see Fig. 5b). For the higher ambient pressure, the barrel shock position moves closer to the jet axis and the shock density peak values increase from 0.3 × 10^18^ cm^−3^ at 1 mbar to 10^18^ cm^−3^ at 3 mbar.

We believe it is crucial to characterize the gas jets operating at high repetition rates with sufficient sensitivity in the exact experimental conditions, as the occurrence of the barrel shock may happen due to the accumulation of ambient gas if insufficient pumping speed of the vacuum system is employed. This might often be the case when light gases such as He or H_2_ are used.

## Discussion

The new multi-pass probing method presented here shows significant improvement in phase sensitivity of the interferometric measurement, as verified by the characterization of low-density gas jets. All optical elements that comprise the four-pass probing setup used in our measurements were chosen to optimize the spatial resolution of the system. An analysis using ray-tracing simulations with exact experimental geometry and models of optical elements provided by their manufacturer revealed that the modulation transfer function cutoff frequency of the four pass probing setup is 52 lines/mm and the optical resolution as defined by the Rayleigh criterion is 23 µm, which is close to the theoretical diffraction limit of 19 µm. The corresponding depth of field is 250 µm.

The length of the optical path between individual passes of the probe through the object affects the possible temporal resolution of the measurement. In the four-pass setup presented here, the path-length between individual passes was 4 m resulting in the total time it takes light to travel between the first and the fourth pass to be 40 ns. Therefore, a pulsed laser diode with nanosecond pulse duration would be an efficient light source matching this temporal resolution. The temporal resolution should be considered in the design of the setup, mainly if fast-evolving objects are supposed to be characterized with high spatial resolution, to avoid degradation of the spatial resolution by motion blur. The gas jets studied here have characteristic times of the order of milliseconds, so we could use a continuous wave light source and adjust the temporal resolution by setting the exposure time of the camera, which was 0.2 ms in our case.

Note that instead of the shearing Wollaston interferometer a different method to assess the phase distortion of the beam can be used for the presented multi-pass probing setup, e.g. the high-resolution wave-front sensor based on multi-beam lateral shearing interferometry^16,26^ or a shearing interferometer with an air wedge^27^. One can also employ one of the digital holography methods^28^ to reconstruct the field of the probe in the object plane, in particular its phase, without imaging the object on the camera.

## Conclusions

A new optical probing technique with increased sensitivity and nearly diffraction-limited spatial resolution was introduced and validated for the characterization of transparent phase objects. The optical scheme that enables up to four passes through the object was implemented with two relay-imaging arms and polarization switching using passive optical elements. The number of passes could be, however, further increased by employing an active electro-optical polarization switch.

This technique was employed for gas density characterization of low-pressure Ar and He gas jets. The four-pass configuration employed in the tomographic interferometry setup has shown to resolve fine features of gas jets with tailored density distributions typically used for LWFA even for the low-refracting He gas. It also demonstrated that the four-pass interferometer is able to resolve the onset of barrel shock formation in nonzero ambient pressure, which can be expected during high-repetition rate operation of gas jets. This is an important capability as most of the applications of both laser-driven X-ray sources and laser particle acceleration demand for increased repetition rates.

Our multi-pass probing method has demonstrated to increase phase-sensitivity in a transmission measurement setup. Multiple interactions with the object can, in general, also increase the effect of the object on probe intensity or its polarization in both transmission and reflection configurations. Thus, we believe the method is suitable to increase the contrast and sensitivity in phase, absorption/reflectivity, or polarization measurements of objects or phenomena that are evolving with characteristic time-scales longer or comparable to the time interval between the first and the last pass of the probe.

## Methods

In this section, we provide more details about the experimental setup and data analysis to provide the reader with complete information to enable reproduction our results.

### Experimental setup

The probe beam was generated using a continuous wave 405 nm mono-mode fiber-coupled laser diode, which was collimated by a singlet lens resulting in a 20 mm beam (1/e^2^ diameter). The wavelength is shorter than that of a He–Ne laser to further increase the phase sensitivity because of the higher wavenumber and higher refractive index of neutral gas^16^.

The gas jet is placed in a vacuum chamber, which has two AR coated glass windows. A 3D printed gas nozzle with a circular throat of 0.5 mm diameter and output orifice of 1 mm is mounted on top of the valve. For the measurements of spatially tailored gas density distribution a razor blade was attached 1 mm above the nozzle output. The valve opening time was set to 10 ms. The nozzle was attached to a motorized rotation stage enabling precise turning of the target with respect to the probe beam, which is needed for the multi-projection tomographic measurements.

The key part of the setup was the relay-imaging optical system ensuring the imaging of the object (gas jet) on itself. For this system, we have employed two identical doublet lenses with a focal length of 500 mm in a 4f telescope configuration and a planar mirror in the image plane of the system. The object was thus first imaged on the planar mirror and this image was imaged back by the same 4f system on the object itself while at the same time the beam remained collimated after passing through this system. The 4f system with doublets was used since the aberrations assessment using raytracing simulations showed that it significantly outperforms the one using a biconvex singlet lens and a spherical mirror reported in^16^.

The polarization switching in the four-pass configuration employed a quarter-wave plate optimized for 405 nm and a polarizing beam-splitter with high extinction ratio (> 10 000:1 for 405 nm) to prevent parasitic double-pass signal superposed on the four-pass one.

The method used for evaluation of the wave-front distortion of the probe beam caused by the gas was shearing interferometry using a Wollaston prism. Shearing interferometry^13^ employed with our method benefits from not requiring a reference beam, which simplifies the setup, as the beam path of the multi-pass probing setup becomes rather long. The gas jet was imaged by a biconvex lens with a 300 mm focal length on a CMOS camera with resolution 2048 × 2048 and pixel size of 5.5 µm with 1.15 magnification. The Wollaston prism with a one-degree deviation angle and a linear film polarizer are placed in front of the camera. The relative position of the Wollaston prism to the imaging lens focal plane was set to adjust the density of interference fringes on the camera. On the other hand, rotating the Wollaston prism around the optical axis allowed modification of the fringe orientation.

### Data processing and density reconstruction

The gas density measurement is divided into three steps—recording of single-projection interferogram, phase retrieval with wavelet transform, and 3D gas density calculation by Abel inversion for axisymmetric jets, or tomographic reconstruction from multiple projections for tailored gas distributions.

To analyze the phase shift from the interferograms we employed 2-D CWT^29,30^ followed by a direct maximum ridge detection algorithm^31^. The wavelet transform has improved capabilities in the reconstruction of non-stationary signals when compared to Fourier transform methods, especially with regards to noise ^32^. The downside of using 2D CWT is an increase in computing time. We have used the Morlet mother wavelet, which is a complex sine wave tapered by a Gaussian, defined in two dimensions as

$$\psi \left( {\varvec{x}} \right) = \frac{1}{c}e^{{i{\varvec{\omega}}_{0} \cdot {\varvec{x}}}} e^{{ - \frac{{\left| {A{\varvec{x}}} \right|^{2} }}{{2\sigma^{2} }}}} ,$$

where ${\varvec{x}}$ is a position vector in a 2-D coordinate system, $A$ denotes the anisotropy matrix diag $(\frac{1}{\sqrt \varepsilon },1)$, ${\varvec{\omega}}_{0} = \left( {0,\omega_{0} } \right)$. with $\omega_{0}$ denoting the spatial frequency, $\sigma$ describes the width of the Gaussian envelope. Normalization of the wavelet is ensured by appropriate constant $c$. Finding the best match of translated, rescaled, and rotated wavelet for the particular position of the interferogram allows for precise evaluation of local fringe shift, which represents the probe phase. We use the convenient value $\omega_{0} = 2\pi$ rad/pix and scale the daughter wavelet frequency $\omega = \omega_{0} /a = 2{\uppi }/a{ }$ to the value of the main fringe frequency by setting scale $a$ around fringe period. For our case of fringe period being ~ 6 pix we used a set of scales to cover the neighborhood of 6 by 100 values evenly spaced between 2 and 10. For our fringe tilt of π/4, the wavelet rotation is sampled by 10 evenly spaced values ranging from π/12 to π/2. To discern local fringe features better, we chose fewer oscillations and experimentally set the value of $\sigma$ to 0.75. Lastly, we set anisotropy factor to ε = 0.75 based on results of analysis made with artificial interferograms.

**Figure 6:**
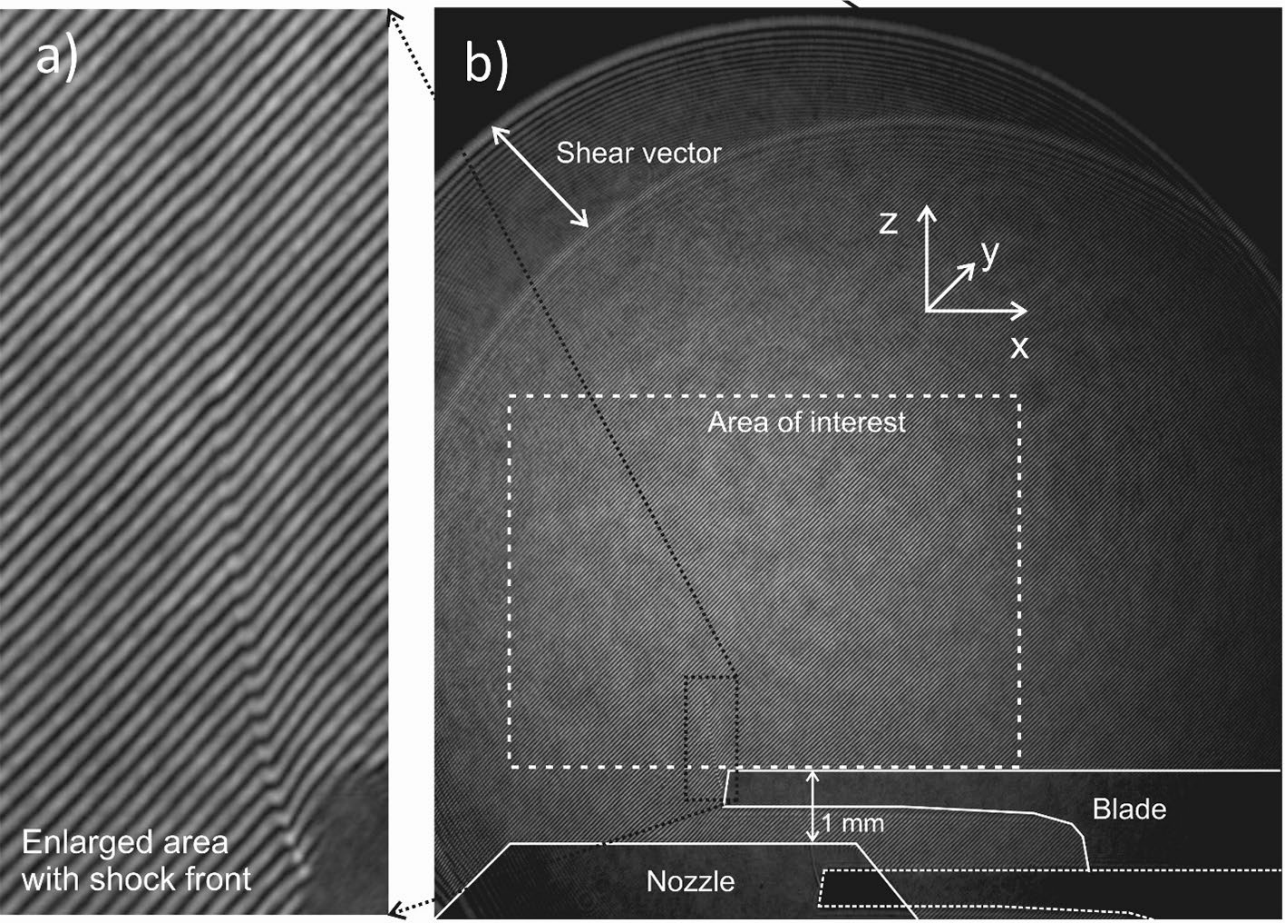
(**a**) Part of the raw interferogram with bent fringes due to the shock front from the blade. (**b**) Raw interferogram with schematics showing the positions of the nozzle and the blade.

The interferogram from a shearing interferometer is a result of the interference of two different sections of the laser beam (Fig. 6)^33,34^. For each individual measurement, we record two interferograms—a signal one with the gas jet target and a reference one without the gas jet and subsequently subtract the retrieved phase maps. This improves the measurement by eliminating possible phase distortions caused by the optical system imperfections.

**Figure 7:**
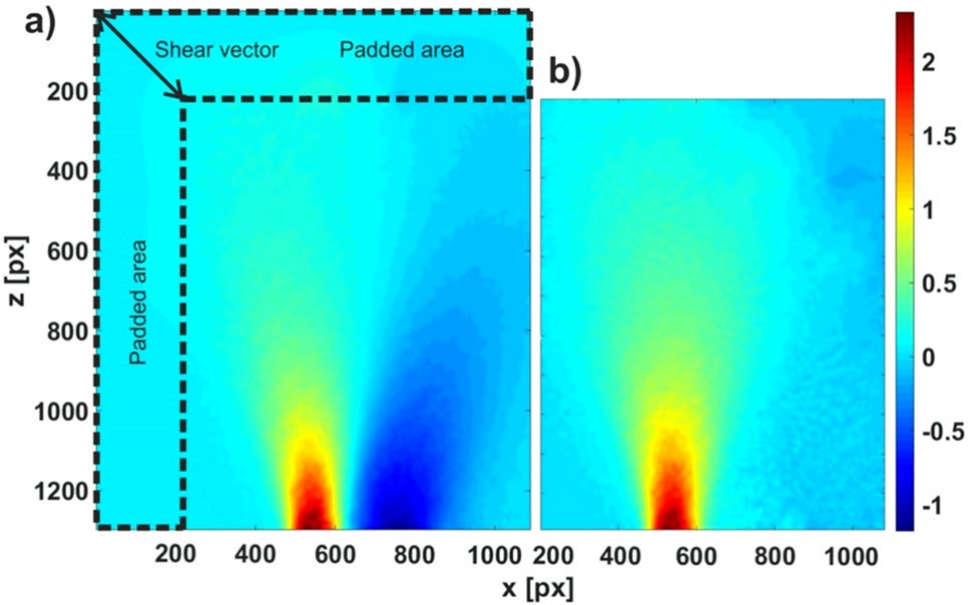
(**a**) Phase map with second Wollaston copy visible as a dark blue region in the right of the jet (Argon, backing pressure 1.6 bar). The area marked by the dotted line is the extension with a size corresponding to the shear vector ${\varvec{s}}$. (**b**) Corrected phase map of the same interferogram.

Even though the separation of the two interfering beams in the Wollaston interferometer is large, the object is rather extended and the two copies of the sheared wavefront might interfere in some regions. To correct for this effect, we have first extended the phase map by a smooth extrapolation in the direction of the shear vector ${\varvec{s}}$ (see Fig. 7) and corrected the phase maps obtained by the CWT to extract the original wavefront. Starting from the top left corner and continuing row by row we added to the value in position $\left( {x,z} \right) + {\varvec{s}}$ to the the value in $\left( {x,z} \right)$.

The depth of field of our imaging system is comparable to the object size and the scattering of the object is low. That is why the resulting phase of the probe could be considered as a geometrical projection of the object in the probe direction. By reducing the depth of field while increasing the spatial resolution of the imaging one would need to take into account the probe beam propagation in the object and apply one of the beam propagation algorithms^35,36^.

For the assessment of the density distribution of gas jets with rotational symmetry, we have used the inverse Abel transform^37^. This method allows the reconstruction of the gas density distribution from a single projection recorded in the direction perpendicular to the axis of symmetry. For objects of arbitrary gas density distributions, e.g. shock fronts or non-rotationally symmetric gas jets, the Abel inversion is not a viable solution. Such objects can only be reconstructed by the acquisition of multiple projections and the use of tomographic reconstruction algorithms. Stacking of the recorded projections together with the help of the Fourier slice theorem^38^, allows for reconstructions of the complete spatial density. Here, we used the Filtered Back Projection (FBP) method for tomographic reconstruction^38^. This method is in practice a discrete implementation of the Inverse Radon transform^39^. We employed a high-pass ramp filter and a Hann filter to eliminate the over-representation of low frequency components and to suppress the low-frequency numerical noise that the FBP is known to induce. This significantly reduces image blurring. We have made 90 projections with a step of 2 degrees for all the tomographic measurements presented here. According to the analysis reported in^14^, this number of projections will theoretically result in tomographic reconstruction with a relative error of around 1% between the reconstructed and modeled object function.

## Data Availability

The data that supports the plots within this paper and other findings of this study are available from the corresponding author upon reasonable request.
